# Low health-related quality of life in school-aged children in Tonga, a lower-middle income country in the South Pacific

**DOI:** 10.3402/gha.v7.24896

**Published:** 2014-08-20

**Authors:** Solveig Petersen, Boyd Swinburn, Helen Mavoa, Kalesita Fotu, Caroline Tupoulahi-Fusimalohi, Gavin Faeamani, Marjory Moodie

**Affiliations:** 1 Deakin Health Economics, Faculty of Health, Deakin University, Burwood, Victoria, Australia; 2 WHO Collaborating Centre for Obesity Prevention, Faculty of Health, Deakin University, Geelong, Victoria, Australia; 3 Child and Adolescent Psychiatry, Department of Clinical Sciences, Umeå University, Umeå, Sweden; 4 Epidemiology and Global Health, Department of Public Health and Clinical Medicine, Umeå University, Umeå, Sweden; 5 School of Population Health, Faculty of Medical and Health Sciences, University of Auckland, Auckland, New Zealand; 6 Fiji School of Medicine, College of Medicine, Nursing & Health Sciences, Fiji National University, Nuku'alofa, Tonga; 7 Research and Information, Division of Economic Development, Secretariat of the Pacific Community, Suva, Fiji

**Keywords:** adolescent health, child health, community health, Epidemiology, low-income population, mental health, quality of life

## Abstract

**Background:**

Ensuring a good life for all parts of the population, including children, is high on the public health agenda in most countries around the world. Information about children's perception of their health-related quality of life (HRQoL) and its socio-demographic distribution is, however, limited and almost exclusively reliant on data from Western higher income countries.

**Objectives:**

To investigate HRQoL in schoolchildren in Tonga, a lower income South Pacific Island country, and to compare this to HRQoL of children in other countries, including Tongan children living in New Zealand, a high-income country in the same region.

**Design:**

A cross-sectional study from Tonga addressing all secondary schoolchildren (11–18 years old) on the outer island of Vava'u and in three districts of the main island of Tongatapu (2,164 participants). A comparison group drawn from the literature comprised children in 18 higher income and one lower income country (Fiji). A specific New Zealand comparison group involved all children of Tongan descendent at six South Auckland secondary schools (830 participants). HRQoL was assessed by the self-report Pediatric Quality of Life Inventory 4.0.

**Results:**

HRQoL in Tonga was overall similar in girls and boys, but somewhat lower in children below 15 years of age. The children in Tonga experienced lower HRQoL than the children in all of the 19 comparison countries, with a large difference between children in Tonga and the higher income countries (Cohen's *d* 1.0) and a small difference between Tonga and the lower income country Fiji (Cohen's *d* 0.3). The children in Tonga also experienced lower HRQoL than Tongan children living in New Zealand (Cohen's *d* 0.6).

**Conclusion:**

The results reveal worrisome low HRQoL in children in Tonga and point towards a potential general pattern of low HRQoL in children living in lower income countries, or, alternatively, in the South Pacific Island countries.

Ensuring a good life for children is high on the public health agenda in most countries (1). The emphasis has previously been on early childhood and increasing survival rates, while older children generally have been seen as healthy and therefore largely neglected (2). In the school ages, survival rates are comparably high, but recent reports indicate substantial levels of physical and emotional ill-health, which calls for raised awareness of the health and wellbeing in this age group.

Health-related quality of life (HRQoL) focuses on the subjective experience of physical, emotional, and social functioning and wellbeing and thereby captures all the core aspects of health simultaneously (3). Child-HRQoL has been demonstrated as an indicator of health care costs and future illness (4, 5). As such, this outcome has gained an increasing role in guiding health and welfare policies. The concept is however relatively new in the paediatric field. Only since the turn of the century have standardized instruments become available for child assessment. Translations of these instruments for a wider international use are ongoing. Therefore, the knowledge about HRQoL in child populations is still limited.

Investigations of child-HRQoL in general populations, including socio-demographic variations, are particularly limited and the evidence is almost exclusively reliant on data from upper-middle or high-income (higher income) countries, primarily in the West (6). This evidence may not be directly transferable to low- or lower-middle income (lower income) countries, or non-Western cultures. The only previous community-based study of childhood HRQoL in a lower income country (Fiji), reported particularly low HRQoL in this setting (6).

The current study was performed in the Kingdom of Tonga, a lower-middle income country in the South Pacific with a gross national income per capita of USD 4,200 (constant 2005 purchasing power parity) (7). In 2006, the population of Tonga comprised 101,991 inhabitants, 86% of whom lived on the two largest islands of Tongatapu (71%) and Vava'u (15%), close to everyone (98%) was of Tongan ethnicity, and half of the population was below 19 years of age (8). Many Tongans have migrated to New Zealand, and in 2006 a total of 50,478 Tongans lived there, with 80% in or around Auckland, the most populous part of the country (9). The median age of these migrant Tongans was 19 years; 56% were born in New Zealand and the median annual income was NZD 17,500 (~USD 12,248; June 2005) compared to NZD 24,400 (~USD 17,077; June 2005) in the whole New Zealand population.

The objectives of the current study were to investigate the HRQoL of children in Tonga, by age, sex, and living situation, and to compare the HRQoL of children in Tonga to that of children in other countries, including Tongan children living in New Zealand.

## Methods

### Procedure and study populations

This cross-sectional study used data from the Pacific Obesity Prevention in Communities (OPIC) project, which has been described in detail elsewhere (10). In short the OPIC project targeted secondary schoolchildren at a community-level in four countries, including Tonga and New Zealand. In Tonga, all secondary school children on the island of Vava'u and in three districts of the main island of Tongatapu (Kolonga, Houma, and Nukunuku) were invited to participate in the OPIC project, as were all children in six South Auckland high schools in New Zealand. The current study presents data from when the children entered the OPIC project between year 2005 and 2008. The Tongan study site comprised 4,536 children aged 10–19 years (according to the 2006 Tongan Census). Of these, 64% participated in the OPIC project. Due to equipment failure, HRQoL data were only available from 2,164 children (47%). A New Zealand comparison group comprised all children in the OPIC project who identified themselves as Tongans (*n*=830). After written informed consent from the child and a parent, the child filled in questionnaires in the classroom, supported by trained research assistants.

### Measures

*HRQoL* was measured by the adolescent self-report version of the generic Pediatric Quality of Life Inventory™ 4.0 (PedsQL), developed by Dr. James W. Varni (11). The 23 item PedsQL instrument measures overall HRQoL along with the four subdomains: physical (eight items), emotional (five items), social (five items), and school (five items) functioning and wellbeing (the 15 item from the latter three also constituting an overall psychosocial domain). The recall frame is 1 month and items have five response alternatives, ranging from 0) ‘never’, to 4) ‘almost always a problem’. As recommended, responses were transformed onto a 0–100 scale and mean scores were calculated for domains with at least 50% of items answered within the scale. One hundred children had failed to fill in between two and nine PedsQL items, which resulted in missing subdomain scores in 54 children. Due to the multidimensionality of the HRQoL concept, the overall HRQoL and psychosocial scores were only calculated when all associated subdomains could be generated. Higher scores indicate better HRQoL and overall/subdomains scores in the lowest quartile were defined as low.

The questionnaire was translated into Tongan language in accordance with the Linguistic Validation Guidelines set down by the Mapi Research Institute using a double forward and a back translation process (http://www.mapi-institute.com/linguistic-validation). Semantic equivalence and cultural appropriateness of the translated instrument was verified in focus group studies. The PedsQL has demonstrated acceptable reliability and validity in diverse cultural contexts around the world (11–15), and confirmatory factor analyses have revealed factorial invariance across boys and girls (12), age groups (16), socio-economic status (17), and diverse cultural groups, including Asian/Pacific Islanders (18). In the current samples from Tonga and New Zealand, all domain scales demonstrated acceptable internal consistency reliability (Cronbach's *α* 0.7–0.9), and no major floor or ceiling effects [<0.2% scored 0 in a scale; 1.5–15.1% (Tonga) and 2.2–24.8% (New Zealand) scored 100 in a scale].

*Socio-demographic characteristics* were captured by questions about age, sex, ethnicity, family structure [living with one parents, two parents, not with parents (other adult relatives, boarding school, none of these)], and number of people living in the student's home (household size). Residential location (Vava'u and on Tongatapu: Kolonga, Houma, and Nukunuku) was registered at data collection.

### Comparison groups from the literature

Comparison studies from the literature were searched in the PubMed and PsycInfo databases by use of the search terms ‘Pediatric Quality of Life Inventory’ or ‘PedsQL’ (February 2014). To qualify as a comparison study, a study had to be community-based, include school-aged children, measure self-reported HRQoL using the PedsQL, and present both total and subdomain scores. Only one study per country was selected, taking into account comparability to the sample in Tonga, and representativeness of the country under study (using conventional criteria for representativeness, e.g. a national study was preferred over a local study).

### Analyses

Statistics were performed using the Predictive Analytics Software, version 18. Internal consistency of the PedsQL domain scales was estimated by Cronbach's coefficient alpha, with values ≥0.70 considered acceptable for group comparisons and values ≥0.90 acceptable for individual comparisons (19). Summary statistics were estimated for all PedsQL domains, and mean scores were descriptively compared between Tonga and the other countries. In the comparisons between Tongans in Tonga and New Zealand, children in Tonga with a non-Tongan ethnicity were omitted. Due to the HRQoL measure having an ordinal-scale basis, the non-parametric Mann Whitney U test was used to detect HRQoL differences between socio-demographic groups in Tonga, i.e. age: <15 vs. ≥15 years; sex; residential location (Tongatapu vs. Vava'u); family structure (living with two parents vs. other arrangements); and household size (1–5 vs. 6–10 and 11–16 persons). Associations between a low HRQoL in Tonga and age or sex groups were also tested in univariate and multivariate logistic regression models, with HRQoL overall/subdomain scores in the lowest quartile (yes vs. no) as the dependent variable, age group/sex as the independent variable (or potential confounder when not an independent variable), and ethnicity (Tongan vs. others), residential location (Vava'u or Tongatapu), family structure (living with two parents vs. others), and household size as potential confounders. Level of significance was set at 0.05. The size of differences was estimated by Cohen's *d* with values ≥0.2 considered meaningful but small, and values ≥0.5 and ≥0.8 of medium and large sizes, respectively (20).

### Ethics

Ethics approval was obtained from the Tonga National Health Ethics Research Committee, Deakin University Human Research Ethics Committee, and the University of Auckland Human Participants Ethics Committee.

## Results

### Participant characteristics in Tonga

The 2,164 participants in Tonga had a mean age of 15.0 years (standard deviation, SD: ±1.9 years), comprised slightly more girls than boys, and were almost exclusively of Tongan descendent (Table 1). The sample was equally distributed between Tongatapu and Vava'u, seven out of 10 children lived with both parents, and the mean household size was 7.4 persons (SD: ±3.1 persons).

**Table 1:** Basic characteristics of the participants in Tonga

	*N*	%
All children	2,164	100.0
Sex		
Boys	989	45.7
Girls	1,174	54.3
Age (years)		
11	110	5.1
12	231	10.7
13	372	17.2
14	353	16.3
15	362	16.7
16	358	16.5
17	241	11.1
18	137	6.3
Age group (years)		
<15	1,066	49.3
≥15	1,098	50.7
Ethnicity		
Indigenous	2,123	98.1
Others	41	1.9
Residential location		
Tongatapu	1,077	49.8
Vava'u	1,087	50.2
Family structure during the school week		
Living with		
2 parents	1,469	67.9
1 parent	309	14.3
Other adult relatives	224	10.4
At boarding school	103	4.8
None of the above	54	2.5
Unknown	4	0.2
Household size		
1–5 persons	558	25.8
6–10 persons	1,221	56.4
11–16 persons	279	12.9
Unknown	106	4.9

### HRQoL in Tonga

During the 4 weeks preceding the survey, 77% of the schoolchildren in Tonga had experienced at least one frequent (often or almost always) HRQoL problem and 51% reported more than one frequent problem, within at least one of the physical, emotional, social, and school subdomains.

#### Age, sex, and living situation

Younger schoolchildren below the age of 15 years experienced a small but meaningfully lower HRQoL than older children, mainly due to a lower physical and social functioning and wellbeing (Table 2). Correspondingly, the odds of having a HRQoL in the lowest quartile was 1.5 times higher (95% CI: 1.3–1.8) in younger than older children, and younger children had 1.6 and 1.5 times higher odds of low physical and social functioning and wellbeing (95% CI: 1.3–1.9 and 1.2–1.8), respectively, while the two age-groups were similar with regard to low emotional, and school functioning and wellbeing. No major changes occurred when adjusting for sex, ethnicity, residential location, family structure, and household size (detailed data available from the authors upon request).

**Table 2:** Self-rated health-related quality of life (HRQoL) and subdomains of HRQoL by socio-demography in 11–18 year old school children in Tonga

						Age group				Sex				Residential location			
		
	All					11–14 years	15–18 years			Girls	Boys			Tongatapu	Vava'u		
			
		Percentiles																					
	
	*N*	25	50	75	Mean (SD)	Mean (SD)	Mean (SD)	*p*	Cohen's *d*	Mean (SD)	Mean (SD)	*p*	Cohen's *d*	Mean (SD)	Mean (SD)	*p*	Cohen's *d*
HRQoL	2,110	58.70	69.57	80.43	69.35 (15.87)	67.59 (16.41)	71.04 (15.15)	**	0.22	69.20 (15.01)	69.51 (16.85)		0.02	69.64 (16.42)	69.07 (15.34)		0.04
Physical functioning and wellbeing	2,164	56.25	71.88	87.50	71.34 (19.60)	68.60 (20.11)	73.98 (18.74)	**	0.28	70.90 (18.33)	71.84 (21.01)		0.05	71.23 (19.85)	71.44 (19.37)		0.01
Psychosocial functioning and wellbeing	2,110	56.67	68.33	80.00	68.20 (16.67)	67.04 (17.17)	69.32 (16.09)	**	0.14	68.22 (16.06)	68.17 (17.38)		0.00	68.62 (17.26)	67.80 (16.08)		0.05
Emotional functioning and wellbeing	2,156	50.00	65.00	80.00	64.78 (21.03)	64.00 (20.98)	65.53 (21.07)		0.07	62.42 (20.64)	67.57 (21.17)	**	0.25	65.75 (21.48)	63.82 (20.54)	*	0.09
Social functioning and wellbeing	2,120	60.00	75.00	90.00	72.97 (20.14)	70.38 (20.99)	75.49 (18.96)	**	0.26	73.67 (19.17)	72.15 (21.24)		0.08	73.00 (20.39)	72.94 (19.91)		0.00
School functioning and wellbeing	2,162	50.00	65.00	80.00	66.70 (19.40)	66.59 (19.86)	66.77 (18.94)		0.01	68.44 (18.79)	64.60 (19.89)	**	0.20	66.76 (19.46)	66.64 (19.36)		0.01

SD: standard deviation. Cohen's *d* specifies magnitude of difference between groups; values of 0.2 nominated as small; 0.5 as medium and 0.8 as large.

* *p*<0.05

** *p*<0.001.

While girls and boys overall experienced similar HRQoL (Table 2) and had similar odds of a low HRQoL, girls reported lower emotional and higher school functioning and wellbeing than boys (small size differences). In line with these results, girls and boys in general had similar odds of a low HRQoL, but girls had 1.5 higher odds of low emotional functioning and wellbeing (95% CI: 1.2–1.8) and boys had 1.5 higher odds of low school functioning and wellbeing (95% CI: 1.2–1.8) but also 1.2 higher odds of low social functioning and wellbeing (95% CI: 1.03–1.5), while the odds of low physical functioning and wellbeing were similar in boys and girls. Adjusting for other socio-demographic factors did not alter the results significantly (detailed data available from the authors upon request).

HRQoL was similar in children living in Tongatapu and Vava'u (Table 2), in children living with both parents as opposed to other family structures, and in children living in small (1–5 person) versus larger households.

### International comparison (comparison groups from the literature)

Community studies of school-aged children were identified from 19 countries in North and South America (21, 22), Europe (14, 23–29), the Middle East (15, 30), Asia (13, 31–34), Australia (35) and the Pacific (6): 12 high-income countries, six upper-middle income countries and one lower-middle income country (Table 3) (6, 13–15, 21–35).

**Table 3:** Basic characteristics of the community-based comparison group

Country	Sample	*N*	Age (years)	Income classification^a^
Australia (35)	Regional school-based	2,890	11–18	High
Austria (23)	Regional school-based	1,185	8–12	High
Japan (13)	Regional school-based	922	6–18	High
Finland (24)	Regional school-based	1,033	9–10	High
Greece (25)	National school-based	645	8–12	High
Korea (33)	Regional school-based	1,425	8–18	High
Norway (26)	Regional school-based	425	13–15	High
Netherlands (14)	Regional school-based	185	13–18	High
Singapore (32)	School-based	1,249	11–18	High
Spain (27)	Regional school-based	511	9–17	High
United Kingdom (28)	Regional school-based	1,034	8–18	High
USA (21)	Regional household (low-income sample)	5,972	5–16	High
Brazil (22)	Regional school-based (low-income sample)	180	5–18	Upper middle
China (Taiwan) (34)	Regional school-based	519	M 10.5 (SD 1.1)	Upper middle
Iran (15)	Regional school-based	1,188	M 15.7 (SD 1.2)	Upper middle
Jordan (30)	National school-based	150	8–13	Upper middle
Thailand (31)	Regional school-based	2,086	8–15	Upper middle
Serbia (29)	School-based	238	8–18	Upper middle
Fiji (6)	Regional school-based	8,947	12–18	Lower middle

Numbers in brackets after the country refers to the reference list.

a World Bank classification.

**Fig. 1:**
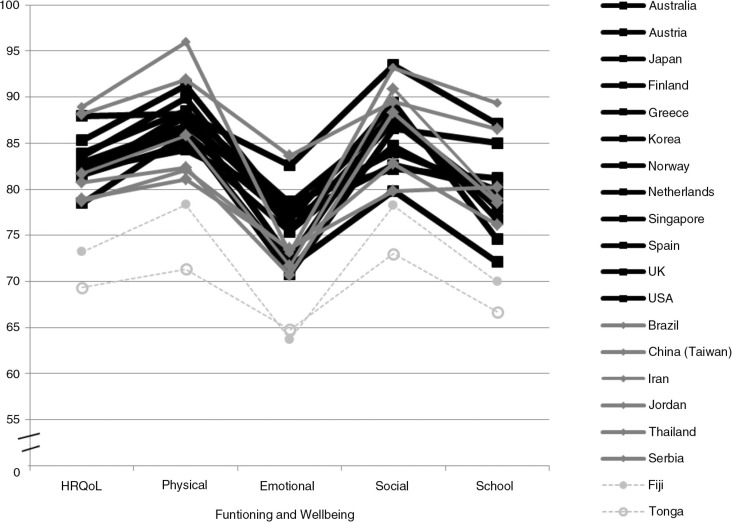
Comparison of self-rated, health-related quality of Life (HRQoL) and subdomains of HRQoL between community based samples of school-aged children in Tonga (grey stippled line, open circle) and in 12 high-income countries (black lines), six upper-middle income countries (grey lines), and one lower-middle income country (grey stippled line, closed circle) (adapted from Refs. 6, 13–15, 21–35). HRQoL is assessed by the PedsQL 4.0 in all samples.

The children in all comparison countries reported better HRQoL than children in Tonga (Fig. 1). The high-income and upper-middle income groups had mean HRQoL scores of 82.8 (SD: ±11.5) and 82.6 (SD: ±11.2), respectively, which was an average of 13 points higher than in Tonga (mean 69.4; SD: ±15.9). Thus, the children in Tonga had a large-size lower HRQoL than the children in the high-income group [mean Cohen's *d* 1.0 (SD: ±0.2); range Cohen's *d* 0.7–1.4] and upper-middle income group [mean Cohen's *d* 1.0 (SD: ±0.4); range 0.6–1.6]. Children in the lower income country Fiji, reported a 4 point higher HRQoL than the children in Tonga (Cohen's *d* 0.3). As for the HRQoL subdomains, there was a general pattern across countries of higher physical and social than emotional and school wellbeing and functioning. In all domains, the children in Tonga reported lower functioning and wellbeing than children in the other countries. The only exception was that children in the two lower income countries, Tonga and Fiji, expressed similar levels of emotional functioning and wellbeing.

### Comparison of Tongan children in Tonga and New Zealand

The 830 Tongan participants living in New Zealand had a mean age of 15.5 years (SD: ±1.6 years) and comprised similar proportions of boys and girls (49% vs. 51%). As in Tonga, seven of 10 Tongan children in New Zealand lived with both parents; 6 of 10 in households of between 6 and 10 members. Of the 560 students who reported country of birth, 419 (75%) were born in New Zealand.

The Tongan children in New Zealand had a medium- to large-size higher HRQoL than the Tongan children living in Tonga (Table 4). This was due to higher functioning and wellbeing across the four physical, emotional, social, and school subdomains in both younger and older children, and in both girls and boys.

**Table 4:** Comparison of health-related quality of life (HRQoL) and subdomains of HRQoL, in 2,123^a^ Tongan secondary school children in Tonga and 830 Tongans in New Zealand (NZ), by age groups and sex

		HRQoL	Physical	Emotional	Social	School
All						
Tongans in Tonga (1)	Mean (SD)	69.38 (15.78)	71.36 (19.54)	64.79 (20.95)	73.03 (20.09)	66.74 (19.38)
Tongans in NZ (2)	Mean (SD)	78.74 (13.76)	82.29 (15.97)	76.61 (19.81)	82.21 (17.86)	71.63 (18.68)
1 vs. 2	*p*	0.000	0.000	0.000	0.000	0.000
	1<2 (%)	11.9	13.3	15.4	11.2	6.8
	Cohen's *d*	0.63	0.61	0.58	0.48	0.26
Age 11–14 years						
Tongans in Tonga (1)	Mean (SD)	67.71 (16.32)	68.73 (20.02)	64.07 (20.94)	70.55 (20.95)	66.66 (19.84)
Tongans in NZ (2)	Mean (SD)	79.01 (13.75)	82.84 (14.84)	77.11 (19.68)	80.46 (18.78)	73.34 (18.26)
1 vs. 2	*p*	0.000	0.000	0.000	0.000	0.000
	1<2 (%)	14.3	17.0	16.9	12.3	9.1
	Cohen's *d*	0.75	0.80	0.64	0.50	0.35
Age 15–18 years						
Tongans in Tonga (1)	Mean (SD)	71.01 (15.08)	73.93 (18.71)	65.49 (20.96)	75.45 (18.92)	66.83 (18.93)
Tongans in NZ (2)	Mean (SD)	78.55 (13.77)	81.89 (16.72)	76.26 (19.91)	83.45 (17.09)	70.41 (18.90)
1 vs. 2	*p*	0.000	0.000	0.000	0.000	0.000
	1<2 (%)	9.6	9.7	14.1	9.6	5.1
	Cohen's *d*	0.52	0.45	0.53	0.44	0.19
Girls						
Tongans in Tonga (1)	Mean (SD)	69.34 (14.82)	71.03 (18.17)	62.46 (20.51)	73.86 (19.03)	68.60 (18.75)
Tongans in NZ (2)	Mean (SD)	78.01 (13.90)	79.97 (16.42)	75.89 (19.74)	82.23 (18.00)	72.59 (18.46)
1 vs. 2	*p*	0.000	0.000	0.000	0.000	0.000
	1<2 (%)	11.1	11.2	17.7	10.2	5.5
	Cohen's *d*	0.60	0.52	0.67	0.45	0.21
Boys						
Tongans in Tonga (1)	Mean (SD)	69.43 (16.86)	71.73 (21.03)	67.50 (21.16)	72.08 (21.24)	64.55 (19.86)
Tongans in NZ (2)	Mean (SD)	79.49 (13.58)	84.67 (15.14)	77.35 (19.87)	82.19 (17.75)	70.64 (18.88)
1 vs. 2	*p*	0.000	0.000	0.000	0.000	0.000
	1<2 (%)	12.7	15.3	12.7	12.3	8.6
	Cohen's *d*	0.66	0.71	0.48	0.52	0.31

SD: standard deviation. Cohen's *d* specifies magnitude of difference between groups; values of 0.2 nominated as small; 0.5 as medium and 0.8 as large.

a Children of non-Tongan ethnicity excluded.

## Discussion

In this community-based sample of secondary schoolchildren in Tonga, a lower-middle income country in the Pacific, HRQoL was similar overall in girls and boys, but somewhat lower in children below, than above, 15 years of age. Children in Tonga reported markedly lower HRQoL than children in upper-middle and high-income countries, including Tongan children living in New Zealand, a high-income country in the same region. The HRQoL difference was small between children in Tonga and Fiji, another country in the Pacific region, but with a similar income level as Tonga. Lower HRQoL in Tonga was due to persistently lower functioning and wellbeing within all studied aspects of HRQoL (physical, emotional, social, and school functioning and wellbeing).

The international comparisons of child-HRQoL were based entirely upon population-based studies, almost exclusively performed in school settings and all assessing HRQoL by the same method. There are, however, other methodological inconsistencies across studies, for instance regarding sample selections. Therefore, the emphasis should be on patterns across studies rather than detailed between-country analyses. The stable pattern of lower HRQoL in Tonga vs. the international comparison group is notable. Similar patterns are found in the literature for depressiveness and bullying. The Tongan national Health Behaviour and Lifestyle of Pacific Youth survey (HBLPY-2000), reported that three in four 11–16 year olds felt unhappy, sad or depressed, 40% to a degree that was ‘almost more than they could take’ and 6 in 10 were bullied/had bullied others (36). This Tongan prevalence of depressiveness and bullying exceeded that in 38 higher income countries surveyed in similar studies in Europe/North America and New Zealand (37, 38).

Clinical studies have reported HRQoL mean PedsQL scores between 70.4 and 82.5 in school-aged patients with asthma, cancer, cardiac disease, diabetes, end-stage renal disease, gastrointestinal conditions, obesity, psychiatric disorder, and rheumatic disease (28, 39). This suggests that the HRQoL in the children in Tonga (mean score 69.4) was not only markedly lower than that of children in other countries, it also reached levels as low, or lower than, that of child patients with serious chronic conditions.

Low HRQoL in the latter part of childhood is not only troublesome at the immediate period. At this life stage, extensive physical and psychological changes occur, self-identify is stabilized, and skills are learned that are critical for success when meeting the challenges and responsibilities of adulthood, including the preparation for a future vocation (40, 41). A low HRQoL may negatively influence these developmental processes and thereby diminish the child's opportunity to develop at his/her full potential, reducing also the prospect of a future good life and valuable contributions to society.

The current study cannot establish causal pathways, but potential explanations can be speculated on. The consistently lower HRQoL in the children living in Tonga, across all studied aspects of HRQoL, may indicate pervasive and/or multifactorial causes. The marked contrast to the New Zealand Tongan children, again across all studied aspects of HRQoL and independent of age and sex may suggest causes related to specific conditions for children living in Tonga, rather than specific ethnic effects.

The small difference in child-HRQoL between Tonga and the lower income country (Fiji) versus the large difference between Tonga/Fiji and the higher income countries, points towards explanations related to country-level income and economic development. This is further highlighted by the comparably lower HRQoL in children in Tonga versus Tongan children living in the lower socio-economic strata in New Zealand (9). Also income inequality, a factor which is known to increase the risk of numerous health problems, (42) is similar in Tonga and Fiji, but higher in these two countries than in all but one (Brazil) of the other comparison countries (43, 44). Thus both absolute and relative income may be of importance when explaining the current findings.

A potential cultural explanatory factor may be related to violence exposure. Whilst there is a total ban on corporal punishment in New Zealand, and several of the comparison countries, this is not the case in Tonga and Fiji where corporal punishment is common (36, 45, 46). Child corporal punishment has been associated with physical ill-health and injuries, short- and long-term mental problems, and impaired relations with parents and peers (47). For instance serious injuries and physical fights are more commonly reported in children in Tonga and Fiji, than in many Western, higher income countries, including New Zealand (37, 48, 49).

The current similar overall HRQoL by sex, but lower emotional scores and higher school scores in girls have also been shown by others (22, 25, 26), while the particularly low HRQoL amongst 11–14 year olds, contradicts the literature (21, 28) and warrants further investigation. One contributing factor may be a possible higher exposure or vulnerability to violence in younger schoolchildren than older ones (36).

The study has both strengths and limitations. The use of a standardized HRQoL instrument with demonstrated sound psychometric properties internationally and across different cultural groups, along with the large sample size, including 15% of all secondary schoolchildren in Tonga (8) probably makes the Tonga data among the best available for lower income countries. Another strength is the parallel inclusion of Tongan children living in New Zealand, a high-income country in close geographic proximity. However, the selection procedure does not secure country-level representativeness, and the participant group in Tonga comprised more children ≥15 years of age than what is seen in Tonga overall (50% vs. 38%) (8). Given that children ≥15 according to our results have somewhat higher HRQoL than those below 15 years (mean 71.04 vs. 67.59), this may have caused a marginally higher HRQoL in our population in Tonga compared to that of Tonga at large. The socio-economic context of the participants in Tonga may on the contrary have influenced HRQoL in the opposite direction. In the study-area, an estimated 70% of the adults had no minimum qualifications, compared to 61% in the whole of Tonga (8), and residence in low socio-economic districts has been related to lower child-HRQoL (50). There were however also several points of similarity between the participant group in Tonga and the whole Tongan population; for instance, similar ethnic and sex distributions (97% vs. 98%, and 54% vs. 50%, respectively) (8), and identical prevalence of health complaints in the study districts and in Tonga at large (8). Finally it should be mentioned that although the PedsQL has demonstrated evidence of factorial invariance across diverse cultural groups (18), more extensive investigations of cross cultural invariance are needed to rule out the possibility that the differences between countries are influenced by the measurement properties of the PedsQL instrument.

In conclusion, children in Tonga seem to experience worrisome low levels of HRQoL, which is of considerable concern as it may have both immediate negative impact and also track into adulthood with negative individual and societal long-term consequences. The results may also signal a potential general pattern of low child-HRQoL in lower income countries, or in South Pacific Island countries, and the country of residence seems to be more closely related with HRQoL than cultural group-belonging. This points out a need for raised awareness about how to support school-aged children's functioning and wellbeing, a need which may be particularly evident in lower income countries. However, the current study is only the second investigation of children's HRQoL in a lower income country, both performed in the Pacific region. Therefore, the results and its potential causes should be further validated within Tonga and also in lower income countries outside the South Pacific region.
